# Study on quality enhancement during cigar tobacco fermentation by *Staphylococcus nepalensis*: insights into microbial community, volatile substances and sensory evaluation

**DOI:** 10.3389/fmicb.2025.1526178

**Published:** 2025-02-11

**Authors:** Qi Pei, Xue Jiang, Zhengqin Li, Hong Xu, Mingyong Xie, Tao Xiong, Zhanggen Liu

**Affiliations:** ^1^State Key Laboratory of Food Science and Resources, Nanchang, China; ^2^College of Food Science and Technology, Nanchang University, Nanchang, China; ^3^International Institute of Food Innovation International, Nanchang University, Nanchang, China; ^4^International Institute of Food Innovation Co., Ltd., Nanchang University, Nanchang, China

**Keywords:** bacterial community, cigar flavor, *Staphylococcus nepalensis*, inoculated fermentation, sensory qualities

## Abstract

**Background:**

The fermentation characteristics of cigar tobacco leaves are closely influenced by the bacterial strains present during the process. This study aims to explore the relationship between bacterial communities and flavor, as well as the impact of key bacterial species on the overall quality of cigars.

**Result:**

The results showed that *Staphylococcus nepalensis* was the dominant bacteria during the fermentation process. Correlations between bacterial communities and flavor revealed that *Staphylococcus nepalensis* was positively correlated with carotenoid degradation products, indicating its potential role in promoting flavor formation. Compared to the control groups, those inoculated with *Staphylococcus nepalensis* showed a significant increase in volatile aroma compounds, particularly megastigmatrienone, and dihydroactinidiolide. Additionally, inoculation with *Staphylococcus nepalensis* resulted in higher consumption rates of reducing sugars and total nitrogen content. However, nicotine levels were lower in the cigars treated with *Staphylococcus nepalensis* compared to the controls. The sensory evaluation further revealed that fermentation with *Staphylococcus nepalensis* notably enhanced the cigars’ aroma quality, reduced irritation, and improved both aftertaste and sweetness.

**Conclusion:**

In summary, the study provides valuable bacteriological resources and a theoretical foundation for optimizing industrial production processes, making it useful for enhancing the quality of cigars in large-scale manufacturing.

## 1 Introduction

The cigar is a special tobacco product, hand-rolled from entirely cigar tobacco leaves (CTLs). The production of cigars involves several stages, including cultivation, air-curing, fermentation, and maintenance. After harvesting, CTLs undergo air-curing and fermentation processes to enhance their quality across various aspects (Tan et al., 2023). Air-curing of CTLs is a technique designed to improve tobacco quality by gradually exposing the leaves to controlled temperatures and humidity levels, thereby accelerating enzymatic and microbial metabolic activity (Zhao et al., 2022). The fermentation phase is critical to the quality of cigars, as it represents the natural continuation of the curing process. During fermentation, CTLs undergo significant improvements, including darkening of color, increased maturity, the elimination of undesirable odors and bitterness, and the development of distinct tobacco flavors. This process ultimately produces a well-balanced product that meets industry standards (Li et al., 2020).

Due to the relatively recent cultivation of cigars in China and the less favorable climate conditions, the aromatic richness of CTLs in China is lower compared to countries like the Dominican Republic and Cuba. Industrial fermentation of cigars typically takes 1–2 years, during which the tobacco undergoes transformation, volatile aromatic compounds accumulate, and a rich flavor profile develops. The inclusion of exogenous additives during fermentation can further enhance the quality of the cigars. Additives such as coffee, cocoa, and other substances are commonly used to increase the alkaloid and sugar content of CTLs, contributing to the mellowness and aromatic depth of the final product (Zong et al., 2023). The microbial community on the surface and inside of CTLs plays a crucial role in degrading large molecules, such as starch and proteins, in the leaves by secreting various enzymes (Liu et al., 2021). Additionally, these microorganisms can produce aroma precursors by promoting the Maillard reaction and breaking down pigments, which contribute to the unique flavor profile of cigars. Microorganisms are present throughout the entire fermentation process and exert significant influence at different stages, shaping the final characteristics of the product (Liu T. et al., 2022). During cigar fermentation, microbial interactions may have influenced the succession of functional microorganisms and microbial communities, thereby affecting CTL transformation and aroma composition (Orland et al., 2018; Zhou, 2021). Previous studies have primarily focused on the changes in the microbial community during CTLs fermentation, with an emphasis on *Staphylococcus* as the bacterial genus (Ye et al., 2021; Zhang G. et al., 2023).

There is significant variability in the microbial composition and dominant flora of CTLs from different origins. The metabolic functions of these microorganisms contribute to the diversity of cigar flavors (Vu et al., 2021; Liu A. et al., 2022). Flavor compounds are central to the quality of cigars, as they determine both the organoleptic characteristics and the stylistic profile of the cigar. Key aroma components, such as Maillard reaction products, carbohydrate degradation products, and neophytadiene, play a crucial role in enhancing the sensory quality of CTLs (He et al., 2023). It has been reported that microorganisms play a crucial role in the formation of volatile compounds during fermentation (Liu et al., 2018). For instance, studies have shown that the dominant bacteria in cigar stack fermentation are *Staphylococcus* and *Corynebacterium*, which influence the composition and succession of microbial communities through their interactions. Additionally, characteristic microorganisms such as *Jeotgalicoccus* and *Geomicrobium* have been found to be positively correlated with the production of dihydro-beta-ionone and isophorone, compounds that enhance the flavor profile of cigars (Wu et al., 2023a).

According to reports, *Staphylococcus* can extensively utilize various carbon and nitrogen sources to degrade proteins and carbohydrates, thereby producing flavor compounds (Sánchez Mainar et al., 2016; Leroy et al., 2017; Hu et al., 2019). During the fermentation process of soy sauce, *Staphylococcus* is the dominant bacterial genus, and the inoculation and fermentation of salt-tolerant *Staphylococcus* strains promote the accumulation of organic acids, such as lactic acid and acetic acid. These organic acids, along with alcohols, can be converted into esters, significantly enhancing the flavor profile of fermented soy sauce (Zhang W. et al., 2024). Moreover, *Staphylococcus* are able to metabolize carbohydrates, convert them into organic acids, and produce characteristic flavor substances that have been widely used in meat fermentation (Aro-Aro et al., 2010). *Staphylococcus* has been shown to dominate the fermentation process of CTLs. Furthermore, *Staphylococcus nepalensis* exhibits strong salt tolerance and can adapt to high-temperature, high-salt conditions during cigar fermentation. It possesses amino acid and lipid metabolism capabilities, which facilitate the conversion of macromolecular compounds and the production of volatile compounds (Ma et al., 2022; Yu et al., 2022). However, there is a lack of research on the inoculation of *Staphylococcu*s for the fermentation of CTLs. We hypothesize that the inoculation of *Staphylococcus nepalensis* during CTL fermentation could enhance cigar quality.

In this study, the microbial community structure during CTLs fermentation was analyzed using high-throughput amplicon sequencing, while volatile flavor compounds in cigars were analyzed using gas chromatography-mass spectrometry (GC-MS). Correlation analysis was then used to explore the effect of dominant bacteria on flavor production. Through the *in vitro* isolation and inoculation of dominant bacteria, their impact on cigar flavor production was assessed, and fermentation performance was evaluated through sensory analysis. This study aims to contribute to the targeted regulation of cigar quality by providing bacteriological resources and a theoretical basis for industrial production.

## 2 Materials and methods

### 2.1 Source and collection of CTLs

The samples for this study were collected from the China Tobacco Corporation of Hainan Province (Haikou, Hainan). The CTLs were categorized into three industrial fermentation stages: the unfermented stage (UFS), the mid-fermentation stage (MFS), and the end fermentation stage (EFS). The UFS CTLs were prepared for fermentation after air-curing, while the MFS CTLs underwent natural fermentation for 1 year, and the EFS CTLs underwent 2 years of natural fermentation. Six samples were randomly collected from the UFS, MFS, and EFS groups to investigate the succession of microbial communities and flavor metabolites during different fermentation stages of CTLs. These samples were subsequently stored at −80°C for no more than 2 weeks before DNA extraction and physicochemical analysis.

### 2.2 Determination of the bacterial community of high throughput amplifiers

Five grams of CTLs were added to 200 mL of 0.85% saline and shaken at 220 rpm for 1 h. The leaves were then filtered out, and the filtrate was centrifuged at 7,000 rpm for 10 min to collect the microorganisms. Amplicon sequencing was performed to sequence the full-length 16S rDNA gene of the microorganisms present in the fermented cigar sample. The sequencing was carried out using amplification primers 338F (5′-ACTCCTACGGGAGGCAGCA-3′) and 806R (5′-GGACTACHVGGGTWTCTAAT-3′) (Jiang et al., 2023). The bacterial amplicons were then used to construct a DNA library, which was subsequently sequenced on the Thermo Fisher Ion S5^™^ XL platform (Thermo Fisher, Waltham, MA, United States) by Personalbio (Shanghai Personal Biotechnology Co., Shanghai, China) (Zhang L. et al., 2023). To obtain high-quality sequencing reads, raw DNA sequencing data were filtered using QIIME2. The quality-filtered sequence data were then clustered into operational taxonomic units (OTUs) at a 97% sequence identity threshold using the QIIME OTU selection workflow. A representative sequence was selected for each OTU using the default settings. The final valid data were aggregated into amplicon sequence variants (ASVs) at the 99% identity level (Huang et al., 2021).

### 2.3 Determination of conventional chemical components

Conventional chemical components in CTLs, including total nitrogen (TN), total sugar (TS), reducing sugar (RS), and nicotine (NIC), serve as important standards for evaluating the quality of CTLs. In this study, continuous flow analysis methods were employed to determine the content of these chemical components, in accordance with tobacco industry standards YC/T61-2002, YC/T159-2019, YC/T217-2007, and YC/T468-2013.

### 2.4 Determination of volatile aroma compound

Aroma compounds are key indicators for evaluating the quality of cigars. The volatile aroma components in CTLs were analyzed using headspace solid-phase microextraction (HS-SPME) combined with gas chromatography-time-of-flight mass spectrometry (HS-SPME-GC-Q-TOF-MS) (Model 8890/7250, Agilent Technologies, United States). A 1.0 g sample of powder and 40 μL of 2-octanol (106.3 mg/L) as the internal standard were placed into a 10 mL glass vial. The mixture was then extracted at 50°C for 30 min using an SPME PAL RSI85 auto-sampler system (CTC Analytics AG, Zwingen, Switzerland) and an Agilent HP-5 MS column (60 m × 0.25 mm × 0.25 μm). Next, the needle was positioned above the sample for 20 min to adsorb the volatile compounds. Following extraction, the fiber was inserted into the GC-MS injection port for thermal desorption at 250°C for 5 min to release the analytes. The GC program had a total runtime of 110 min. The oven temperature was initially set at 40°C for 2 min, then increased to 135°C at a rate of 2°C/min, holding for 5 min. The temperature was further increased to 200°C at a rate of 2°C/min, followed by a 5-min hold. Finally, the temperature was ramped up to 280°C at 10°C/min and held for 2 min. The injector temperature was set to 250°C, with a split ratio of 10:1. The MS parameters were as follows: the ion source was operated with an ionization voltage of 70 eV, and the scanning range was set between 35–400 amu. The ion source temperature was 230°C, and the quadrupole temperature was 150°C. Volatile compounds were identified using the automatic processing module of the NIST17 mass spectrometry library. A semi-quantitative analysis of the volatile compound content was performed by comparing the peak areas of each compound with the internal standard.

### 2.5 Screening and identification of dominant strains

CTLs at different stages of fermentation were sampled into 20 mL of 0.85% NaCl in conical flasks, shaken for 30 min, and then 1 mL of the liquid was aspirated, diluted to an appropriate gradient, and spread evenly on LB agar plates. The plates were incubated at 37°C for 48 h. Single colonies with a staphylococcal appearance were selected and isolated by multiple streaking until purification. The isolated strains were subjected to DNA extraction and 16S rDNA sequencing. PCR was performed using the forward primer 27F (5′-AGAGTTTGATCCTGGCTCAG-3′) and reverse primer 1492R (5′-GGTTA CCTTGTTACGACTT-3′) in a gene amplifier (Eppendorf AG, 22331 Hamburg, Germany) (Zhang L. Y. et al., 2024). The PCR products were then sent to Shanghai Bioengineering Co., Ltd. for bidirectional sequencing and identification. The obtained sequences were compared and analyzed using the NCBI Blast database to determine the species of the strain.

### 2.6 Inoculated fermentation

The strains stored in glycerol tubes were inoculated into a liquid TSB medium and incubated for 24 h. The cultures were then centrifuged at 8,000 rpm for 10 min to collect the cells, which were resuspended in sterile saline to obtain a bacterial suspension. The TSB medium used to culture the strains was prepared with 15 g/L tryptone, 5 g/L soya peptone, 5 g/L sodium chloride, 2.5 g/L potassium dihydrogen phosphate, 2.5 g/L dextrose, and adjusted to pH 7.2. The prepared medium was then sterilized at 121°C for 20 min using an autoclave (Matsushita Health Medical Equipment Corporation, MLS-3751L-PC, China). The strains were then inoculated into 1 kg of CTLs, with the initial cell density of each sample set at 1 × 10^8^ CFU/g. The control groups were sprayed with sterile water. The moisture content of the CTLs was maintained at 30%, with the water naturally absorbed after spraying. The CTLs were then stacked in a constant-temperature and humidity incubator at 37°C and 75% humidity (Shanghai Li-Chen Bangxi Instrument Technology Co., Ltd., HSP-150BE, China), placed in an open sealed bag, and fermented for 21 days. The unfermented sample was designated as control. The samples taken on the 7th, 14th, and 21st days of natural fermentation were labeled CK-7, CK-14, and CK-21, respectively. The samples taken on the 7th, 14th, and 21st days of fermentation with microbial inoculants were labeled N-7, N-14, and N-21, respectively (Zhang et al., 2023a).

### 2.7 Sensory quality evaluation

The fermented CTLs were hand-rolled into cigarettes and stored in an environment with a relative humidity of 69% at 20°C for 36 h to equilibrate the moisture content. In accordance with industry standards, five professional assessors blind-tested the cigars for quality. The evaluated characteristics included aroma quality, aroma intensity, offensive odors, irritation, aftertaste, sweetness, and combustibility.

### 2.8 Data analysis

All samples were analyzed in triplicate, and the results are presented as means ± standard deviation. partial least squares-discriminant analysis (PLS-DA) was performed to evaluate the flavor compound data using SIMCA 14 (demo v.1.0.1). Heatmap analysis of volatile compounds during fermentation was conducted with Tbtools (Toolbox for Biologists) v2.080. A phylogenetic tree of the screened strains was constructed using MEGA 11.0.

## 3 Results

### 3.1 Community structure of eukaryotic microorganisms at different fermentation stages

#### 3.1.1 Diversity analysis

To investigate the structure of bacterial communities at different stages of CTLs fermentation, bacterial populations in various CTL samples were analyzed. The coverage index for all samples reached 0.99, indicating that the sequencing depth was sufficient to capture the full diversity of the bacterial communities present in the cigars.

**Figure 1:**
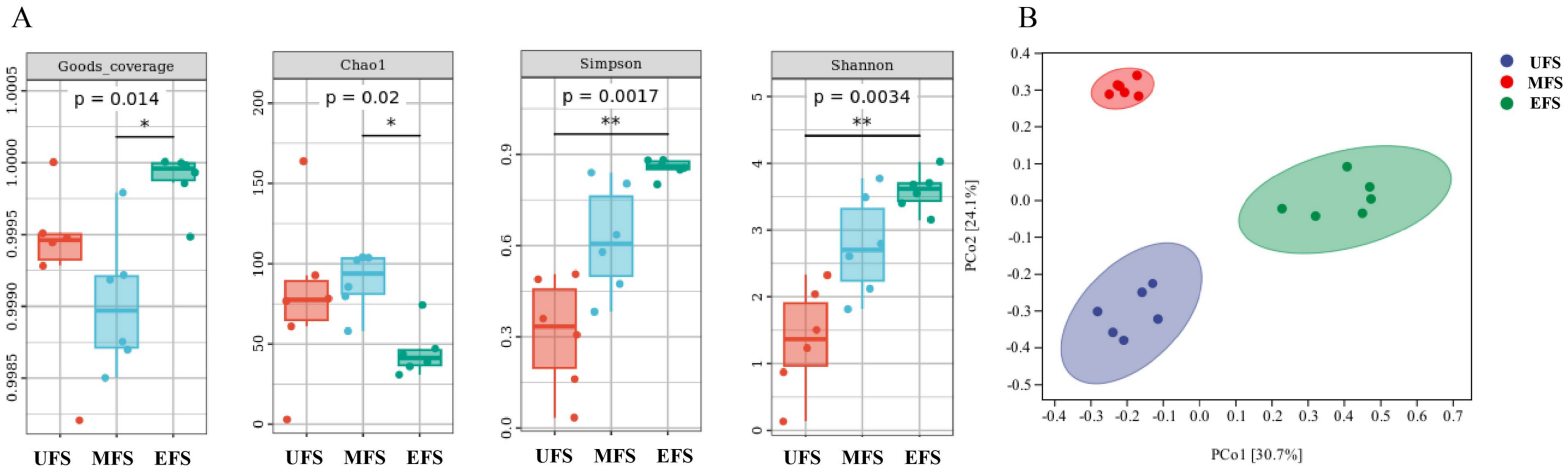
Bacterial alpha diversity **(A)** was determined based on the Goods coverage, the Chaol index, the Simpson index, and the Shannon index. Bacterial beta diversity **(B)** PCoA was measured by unweighted-UniFrac distance.

The Chao1 index was used to assess microbial community richness, while the Shannon and Simpson indices were employed to characterize microbial diversity. The Chao1 index initially increased during fermentation and then decreased, with post-fermentation values being lower than those of the unfermented samples. In contrast, both the Shannon and Simpson indices showed a gradual increase, suggesting that microbial diversity increased over the course of fermentation. This pattern may reflect a reduction in bacterial abundance as fermentation progresses and temperatures rise, with only the dominant bacterial species able to survive the harsher conditions (Figure 1A).

Unconstrained principal coordinate analysis (PCoA) of the unweighted-UniFrac distance revealed that the bacterial communities of CTLs at different fermentation stages formed three distinct clusters (Figure 1B), with high aggregation within each group and clear separation between groups. The samples at the UFS and EFS were nearly overlapping, suggesting that the bacterial communities of these two stages were structurally similar. However, the bacterial communities at the EFS showed a greater degree of change, with more pronounced differences when compared to those at the UFS and MFS.

#### 3.1.2 Bacterial composition and structure

**Figure 2:**
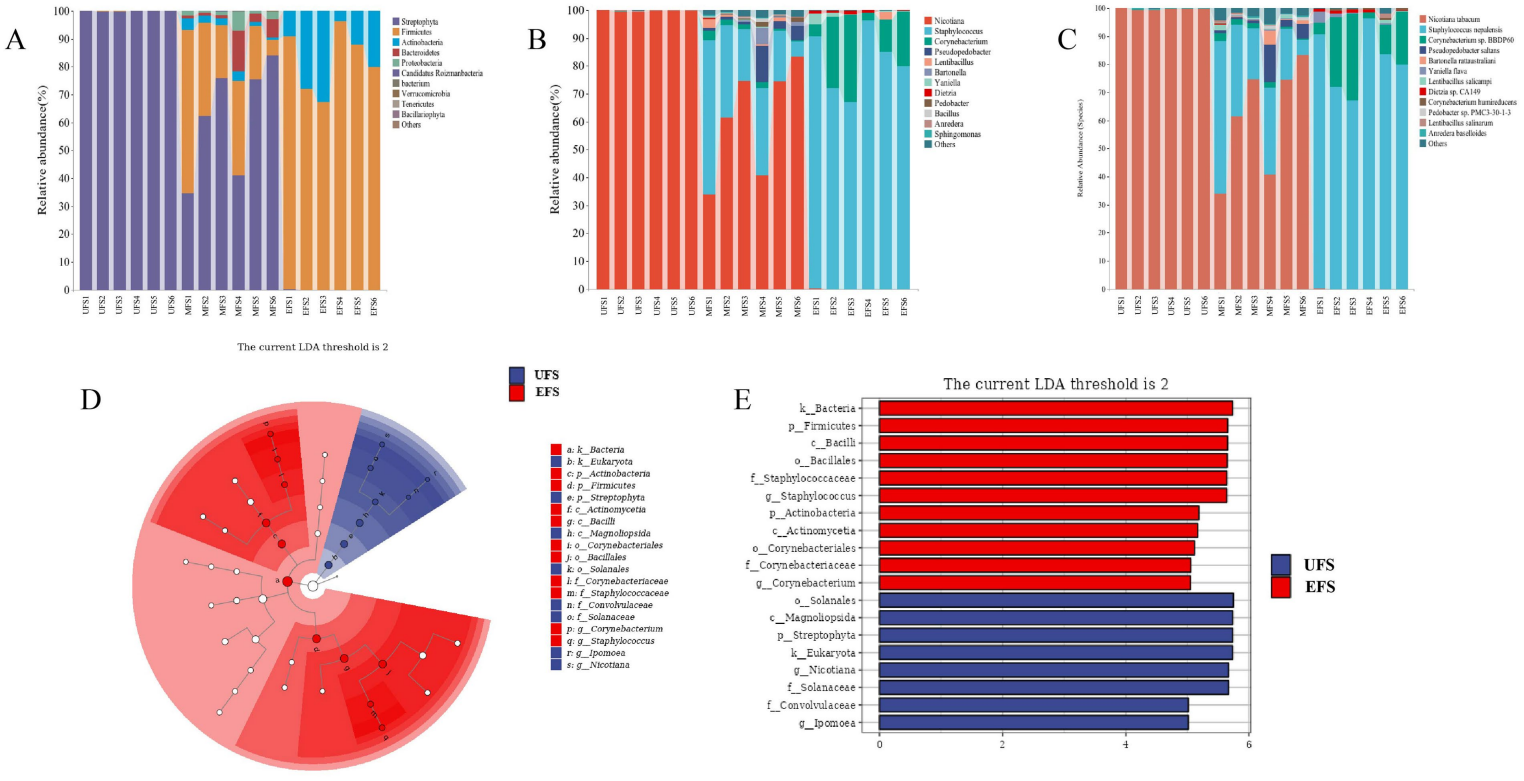
Changes of bacterial at the phyla level **(A)**, genus level **(B)**, and species level **(C)**. LEfSe of the bacteria **(D,E)** during different stages of cigar tobacco leaves fermentation.

The top 10 phyla present throughout the fermentation of CTLs were identified. As shown in Figure 2A, *Firmicutes*, *Actinobacteria*, and *Bacteroidetes* were the dominant phyla during the entire fermentation process, with *Firmicutes* accounting for 82% at the EFS. At the genus level (Figure 2B), fewer microorganisms were detected at the UFS, while a large number of *Nicotiana* and *Ipomoea* were found at this stage. *Staphylococcus* and *Corynebacterium* were the dominant genera at the MFS and EFS, particularly at the EFS, where their abundance reached 81%. This dominance may be attributed to the decreasing moisture content and increasing pH during fermentation, which favors strains with good alkali and salt tolerance, such as *Staphylococcu*s and *Corynebacterium*. This observation aligns with results from previous studies (Wang et al., 2022). At the species level (Figure 2C), *Corynebacterium* sp. *BBDP60* first appeared at the MFS and EFS, with its relative abundance increasing from 1.6 to 15%. The relative abundance of *Staphylococcus nepalensis* also increased throughout the fermentation process, accounting for 0% at the UFS, 26% at the MFS, and 81% at the EFS. Therefore, it is evident that *Staphylococcus nepalensis* was the dominant species during the CTL fermentation process.

#### 3.1.3 Significantly different bacteria during cigar fermentation

To explore the bacteria involved in the fermentation process of cigars, LEfSe analysis was performed to identify significant differences. The results revealed that *Staphylococcus* and *Corynebacterium* were key biomarkers involved in the transformation of inclusions at the EFS (Figures 2D,E). In contrast, two genera, *Nicotiana* and *Ipomoea*, were significantly enriched at the UFS.

### 3.2 Volatile components at different fermentation stages

The flavor compounds in CTLs were analyzed for their volatility using HS-SPME-GC-Q-TOF-MS. A total of 86 volatile substances were detected, including 17 ketones, 10 alcohols, 10 aldehydes, 17 esters, 3 acids, 22 heterocyclic compounds, and 6 other heterocyclic compounds.

**Figure 3:**
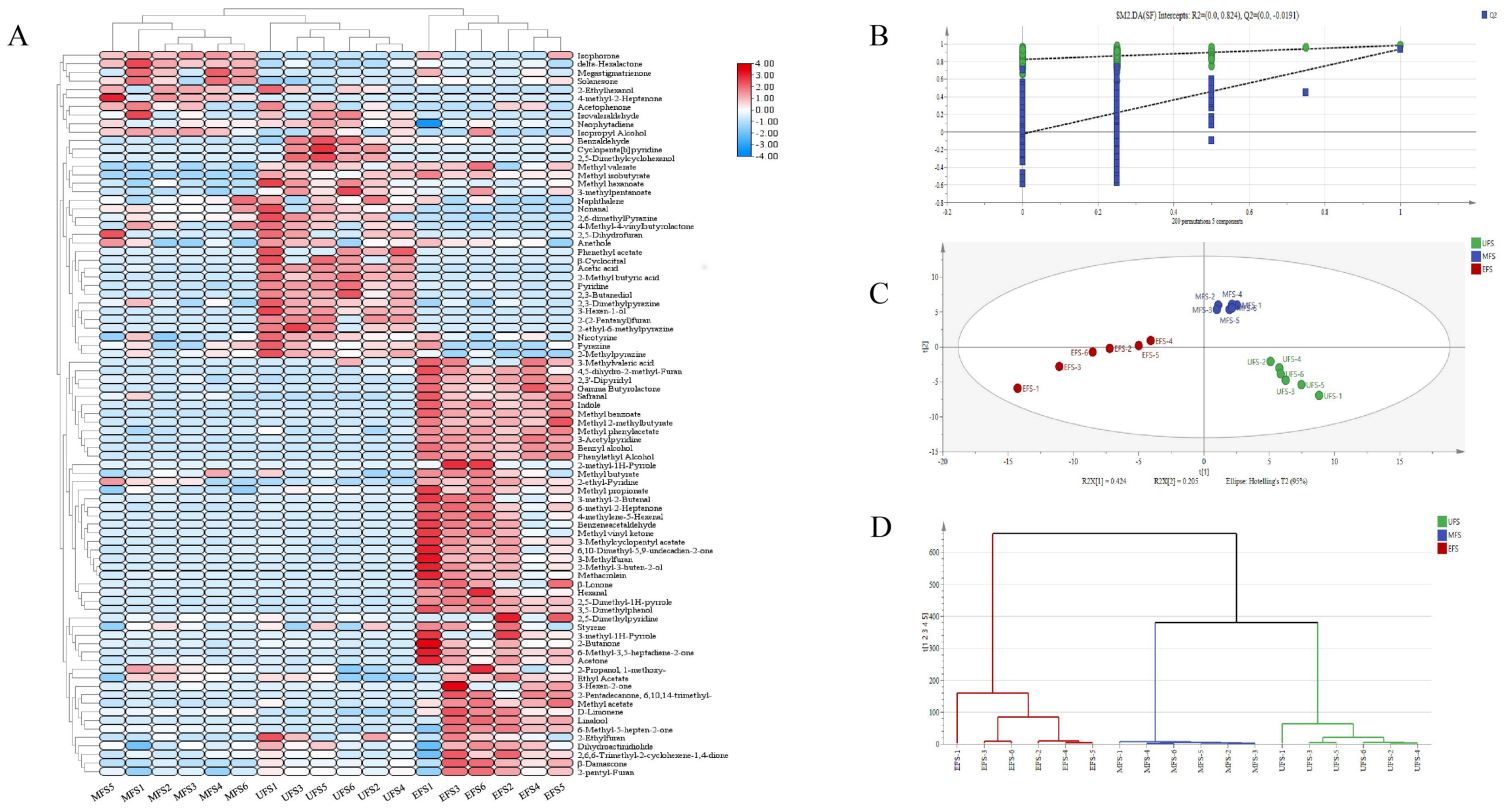
**(A)** Heatmap of changes in volatile compounds at different stages of cigar tobacco fermentation. **(B)** PLS-DA score plots of volatile compounds were generated to analyze their characteristics. **(C)** Permutation tests were performed to compare the three different stages of fermentation. **(D)** HCA plots based on PCA modeling.

The heatmap analysis of volatile aromatic substances at different stages of CTLs is shown in Figure 3A. Compared to the UFS, there was a notable increase in both the diversity and concentration of volatile compounds at the EFS. Among these, ester compounds exhibited significant variability, including dihydroactinidiolide, methyl benzoate, and methyl phenylacetate, which are typically associated with fresh fruit and refreshing aromas. Ketones, primarily resulting from the degradation of carotenoids and cembranoids, were also present. Solanone, a degradation product of cembranoids, imparts a carrot-like aroma that helps mellow the cigar’s overall fragrance (Yun, 2016). Megastigmatrienone, an important flavor compound in CTLs, provides a sweet, dry fruit aroma, which helps reduce off-flavors and enhances the overall quality (Popova et al., 2019). These compounds accumulated during the MFS but gradually degraded during fermentation, likely due to the high temperatures at the EFS, which led to the decomposition of some flavoring substances (Li et al., 2022). Pyrazines are an important class of nitrogen-containing heterocyclic compounds in cigars, contributing to the rich roasted flavor, vanilla, and other pleasant aromas in the smoke, which enhance the overall taste experience (Ashraf-Khorasani et al., 2018). Due to their strong aroma, pyrazines also help mitigate the irritating effect of nicotine-containing smoke on the respiratory tract, making the smoke feel smoother (Alpert et al., 2015). Among them, 2,6-dimethylpyrazine imparts a roasted food aroma, while 2-methylpyrazine offers cocoa and nutty notes. Both of these compounds are found in elevated levels at the UFS stage.

Based on the 86 volatile compounds detected through PLS-DA analysis, the PLS-DA model was validated using 200 response permutation tests, which resulted in *R*^2^ > 0 and *Q*^2^ < 0, confirming that the model has high reliability and good predictive ability (Figure 3B) (Yun et al., 2021). As shown in Figure 3C, the analysis effectively discriminated volatile compounds across different fermentation stages. Additionally, hierarchical cluster analysis (HCA) (Figure 3D) revealed that the CTLs formed three distinct clusters of volatile substances.

### 3.3 Analysis of the correlation between bacteria and volatile substances

**Figure 4:**
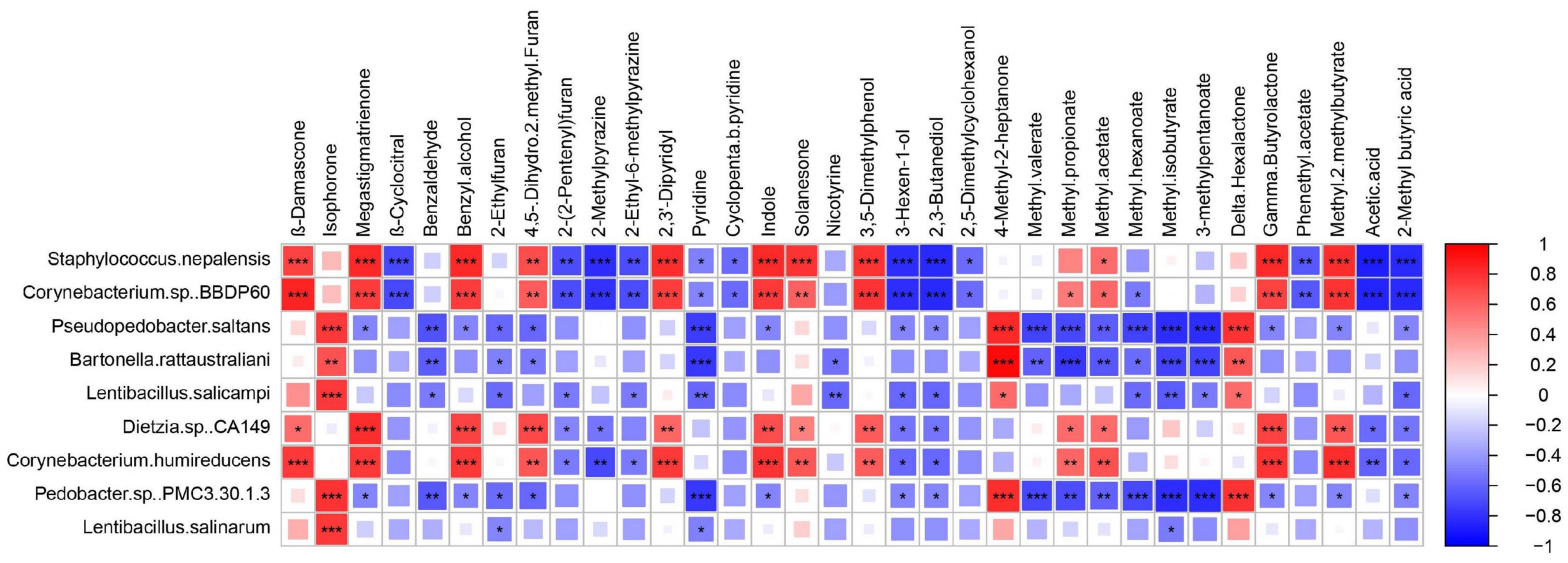
Heatmap analysis of microbial-flavor relationship based on Spearman’s correlation coefficient. Square size and color shade represent correlation coefficient values, and color type represents the positive (red) or negative (blue) correlations. ^*^p < 0.05.

Variable importance in projection (VIP) is used to assess the overall contribution of each variable to the model, with a threshold typically set at VIP >1. A higher VIP value indicates that a volatile compound has a greater impact on the flavor of the cigar sample (Uckun and Selli, 2017). A total of 34 volatile compounds with VIP >1 exhibited significant differences across the three different stages. Spearman’s correlation analysis was performed to examine the relationship between the relative abundance of the top eight bacterial species detected and the 34 volatile flavors (Figure 4).

Based on the above findings, *Staphylococcus nepalensis* was identified as a core functional microorganism in the EFS and found to be strongly correlated with major volatile flavor compounds. It showed a positive correlation with aromatic substances such as megastigmatrienone, solanesone, β-damascenone, benzyl alcohol, and indole, all of which are commonly used in tobacco flavoring to mask unpleasant odors and enhance flavor. This correlation may be linked to the involvement of *Staphylococcus nepalensis* in carbohydrate and amino acid metabolism, which subsequently promotes the production of precursors for these aroma compounds (Hu et al., 2022; Li et al., 2023). In contrast, β-cyclocitral, 2-methylpyrazine, and 2-ethyl-6-methylpyrazine were negatively correlated. Notably, *Corynebacterium glutamicum* had genes involved in the metabolism of aromatic compounds (Mindt et al., 2022). *Corynebacterium* sp. *BBDP60* showed a positive correlation with 4,5-dihydro-2-methylfuran, 2,3-dipyridyl, and indole. Additionally, *Pseudopedobacter saltans* exhibited similar positive and negative correlations with key differential aroma compounds as *Bartonella rattaustraliani*, with both being negatively correlated with most flavor compounds. However, the correlation coefficients derived from statistical analysis only provide predictive insights and do not fully capture the intrinsic relationships between microorganisms and flavor metabolites. The role of *Staphylococcus nepalensis* in promoting cigar flavor compounds requires further verification.

### 3.4 Identification of isolated strains

**Figure 5:**
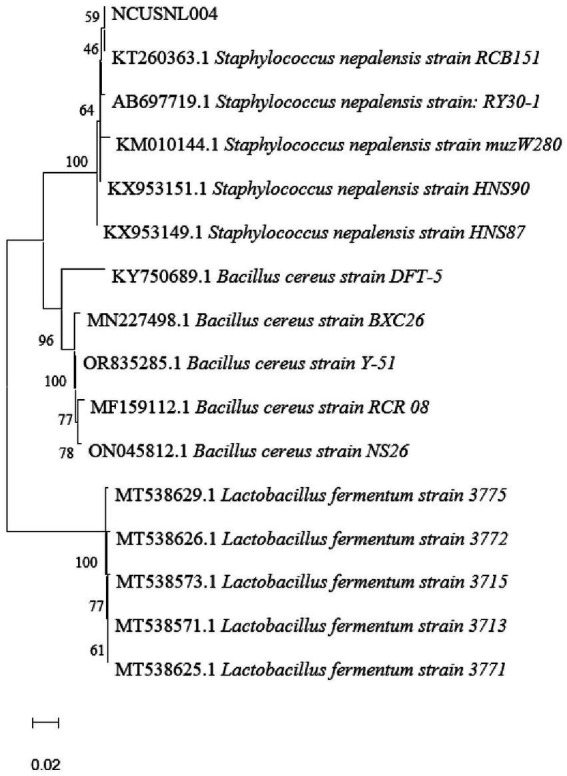
Phylogenetic tree based on 16S rDNA gene sequences, scale bar indicates 0.02 nucleotide substitutions per position.

The 16S rDNA fragments of the strains were amplified to obtain their sequences, which were then analyzed and compared using NCBI BLAST, and a phylogenetic tree construct was constructed by MEGA 11.0 (Figure 5). The results showed that the isolated strain NCUSNL004 clustered with multiple strains of *Staphylococcus nepalensis*, and was identified as *Staphylococcus nepalensis* based on morphological observations as well as physiological and biochemical experiments.

### 3.5 Inoculated fermentation of *Staphylococcus nepalensis*

#### 3.5.1 Changes in conventional chemical composition

**Figure 6:**
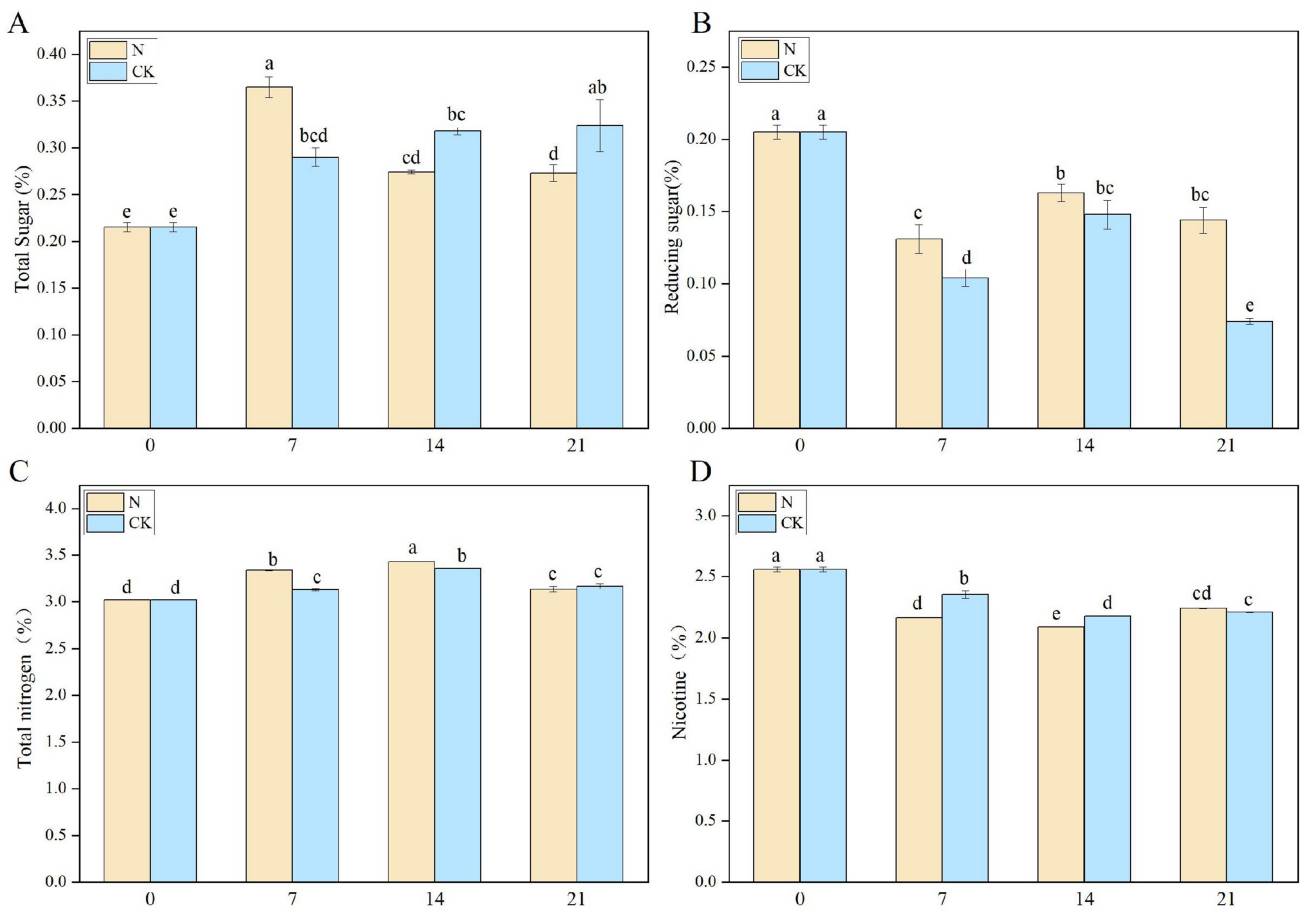
Changes in the chemical composition during bioaugmentation fermentation routines of *Staphylococcus nepalensis*. **(A)** Total sugars content. **(B)** Reducing sugar content. **(C)** Total nitrogen content. **(D)** Total nicotine content. The numbers 0, 7, 14, and 21 indicated the fermentation days. N groups indicates inoculated fermentation with *Staphylococcus nepalensis*, CK groups indicates natural fermentation.

*Staphylococcus nepalensis* was inoculated into CTLs, and the chemical composition was analyzed in both the inoculated fermentation (N) groups and the natural fermentation (CK) groups. Sugar plays a significant role in the sensory quality of CTLs, as it helps reduce the choking sensation and contributes to the formation of various aromatic compounds through the Maillard reaction and pyrolytic processes (Yin et al., 2018). The total sugar (TS) content in the N groups peaked at 0.365% on the 7th day (N-7) but gradually decreased throughout the fermentation process (Figure 6A). In contrast, the TS content in the CK groups gradually increased during fermentation, reaching a maximum of 0.324% on the 21st day (CK-21). Inoculated fermentation accelerated TS production, allowing the starch in the CTLs to be degraded by microorganisms into sugars. After 7 days of fermentation, *Staphylococcus nepalensis* proliferated and utilized the sugars for growth, leading to a rapid decrease in TS content, which is consistent with previous studies (Jia et al., 2023). Reducing sugar (RS) has a positive effect on improving taste absorption and aroma accumulation (Banožić et al., 2020). During the fermentation process, a decreasing trend in RS content was observed (Figure 6B). The RS content in the N groups consistently exceeded that in the CK groups. At the EFS, the N groups showed a decrease of 0.144% (N-21), while the CK group decreased by 0.074% (CK-21).

Total nitrogen (TN) is a key index influencing cigar quality, primarily comprising proteins, free amino acids, and nitrites. The TN content in both the N and CK groups showed an upward trend from days 0 to 14, likely due to the production of amino acids during fermentation (Figure 6C). Inoculation with *Staphylococcus nepalensis* resulted in a higher TN content in the N groups compared to the CK groups. Excessive nicotine (NIC) content can be irritating, and high-quality cigars typically maintain a nicotine content of 1–2%. During the smoking process, nicotine is degraded to azomethine, contributing to the toasted and burnt aroma of CTLs (Silinski et al., 2020). The NIC content decreased over the 0–14 days of fermentation, dropping from 2.56% (control) to 2.09% (N-21) in the N groups, and from 2.56% (control) to 2.21% (CK-21) in the CK groups (Figure 6D). Overall, inoculated fermentation significantly reduced the NIC content.

#### 3.5.2 Changes in volatile flavor compounds

**Figure 7:**
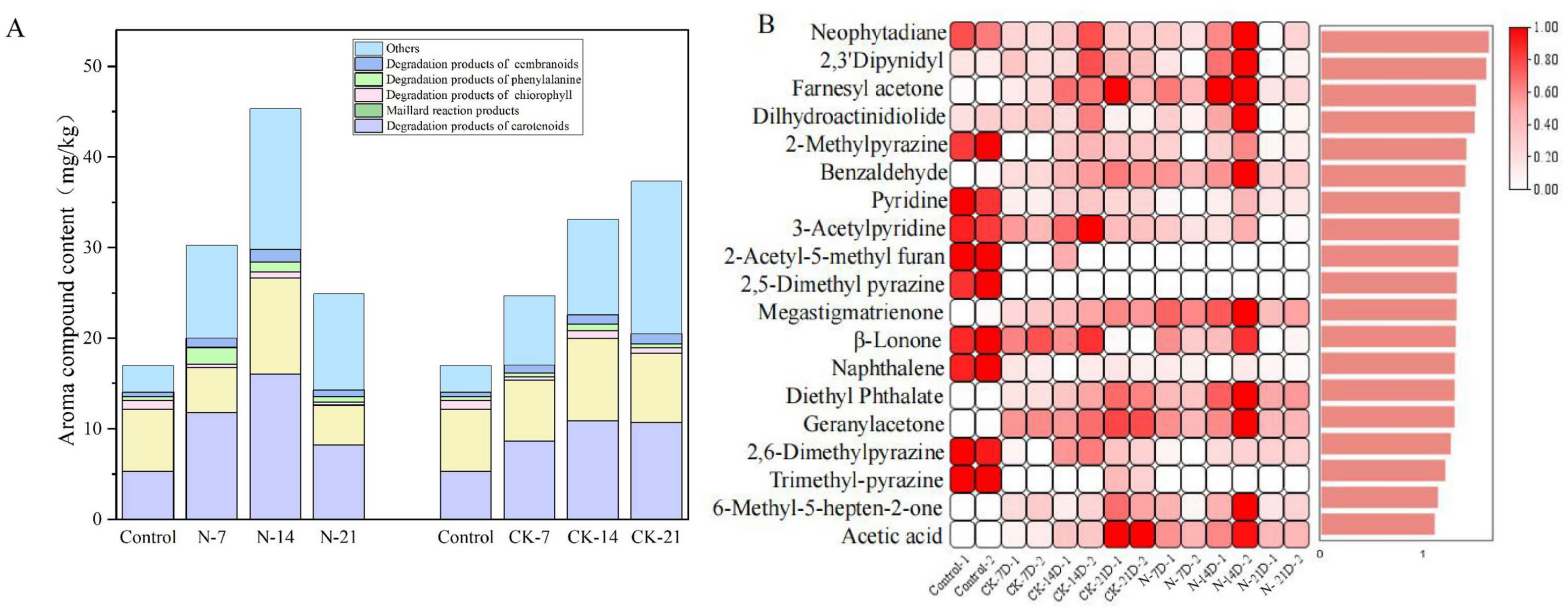
**(A)** Column stacking plot of volatile compound changes during biofortified fermentation of *Staphylococcus nepalensis*. **(B)** Projected importance (VIP) scores for variables based on substance content (VIP >I). N groups indicates inoculated fermentation with *Staphylococcus nepalensis*, CK groups indicates natural fermentation, control groups indicates unfermented (0 days of fermentation). The numbers 7, 14, and 21 indicated the fermentation days.

Figure 7A shows 37 compounds that significantly influenced the flavor of cigars after inoculated fermentation. These compounds were primarily categorized into 9 carotenoid degradation products, 12 Maillard reaction products, 2 chlorophyll degradation products, 1 cembranoid degradation product, 3 phenylalanine degradation products, and 10 other compounds with characteristic aromas. Additionally, the total flavor component content of the CK groups gradually accumulated during fermentation, reaching 37.299 mg/g (CK-21). In contrast, the total flavor component content of the N groups peaked at 45.337 mg/g (N-14) before declining.

To explore the variations in volatile compounds among these samples, 19 compounds were selected as differential volatiles based on a VIP >1 criterion. Among these, 19 compounds showed significant differences across the samples (Figure 7B). Carotenoid degradation products were identified as key contributors to the aroma of CTLs (Popova et al., 2019). Megastigmatrienone, known for its sweet tobacco aroma, enhances the smoke flavor and is an essential flavoring compound in cigarettes (Slaghenaufi et al., 2016; Yang et al., 2016). The content of megastigmatrienone increased significantly after inoculation and fermentation, reaching 8.345 mg/kg (N-14), which is an increase of 7.89 mg/kg compared to the unfermented sample (control). Additionally, dihydroactinidiolide (with a coumarin aroma), geranyl acetone (with a magnolia aroma), and farnesyl acetone (floral aroma) were notably enriched in the N-14 group. Their concentrations increased by 1.595 mg/kg, 0.985 mg/kg, and 0.785 mg/kg, respectively, compared to the unfermented samples (control). These changes may be attributed to the β-carotene degradation capacity of *Staphylococcus nepalensis*, which accelerates the accumulation of these volatile compounds (Wu et al., 2023b).

#### 3.5.3 Analysis of sensory evaluation

**Figure 8:**
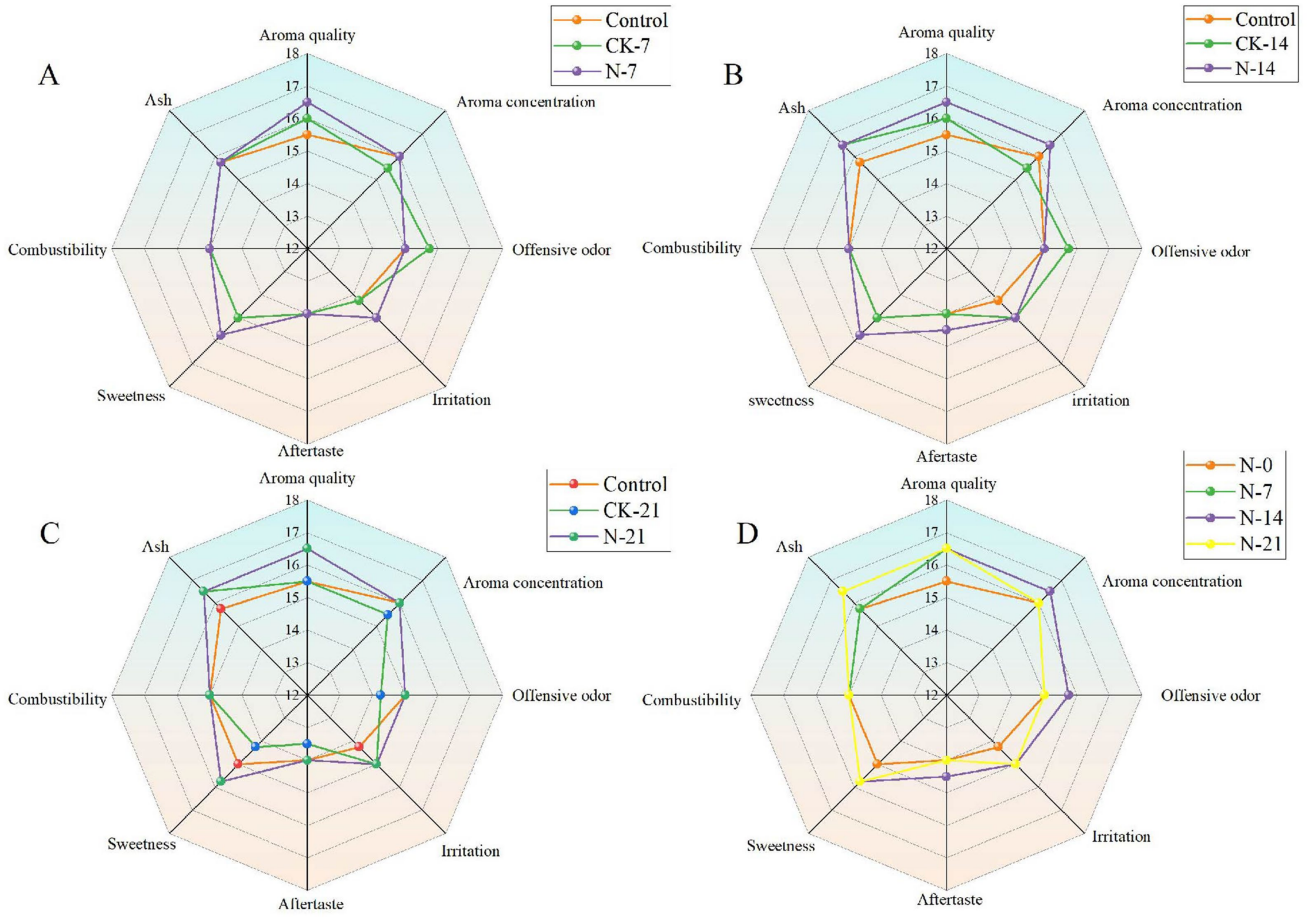
Radar plots of sensory scores of cigars with different cycles of intensive fermentation with *Staphylococcus nepalensis*. **(A)** Seven days of fermentation. **(B)** Fourteen days of fermentation. **(C)** Twenty-one days of fermentation. **(D)** Different fermentation cycles. N groups indicates inoculated fermentation with *Staphylococcus nepalensis*, CK groups indicates natural fermentation, control groups indicates unfermented (0 days of fermentation).

To better assess the impact of functional strains on the quality of CTLs, the effect of different fermentation durations on fermentation efficiency was investigated. Sensory evaluations were performed on samples from 7 days (Figure 8A), 14 days (Figure 8B), and 21 days (Figure 8C) after fermentation. The experimental treatments were divided into three groups: control, CK, and N groups. The results indicated that the overall performance followed the trend of N groups > CK groups > control groups, with variations observed depending on the fermentation time. Specifically, the inoculated fermentation CTLs exhibited a higher concentration of aroma, a mellow and rich flavor, reduced irritation, and increased sweetness. The sensory evaluation results for the different fermentation times are shown in Figure 8D. With the inoculation of fermentation time, the overall organoleptic quality gradually improved, peaking at 14 days before declining at 21 days. On the 21st day of fermentation, however, the quality deteriorated, with a decrease in aroma clarity and brightness, and a less pleasant aftertaste, accompanied by a woody aroma. This may be attributed to over-fermentation.

## 4 Discussion

Microorganisms are widely recognized as playing a crucial role in the quality of cigar tobacco leaves (CTLs) during the fermentation process (Zhang et al., 2023b). High-throughput sequencing studies have shown that the *Staphylococcus* and *Corynebacterium* genera are dominant during CTL fermentation. Furthermore, the relative abundance of this genus continues to increase at different fermentation stages, suggesting its significant role in the fermentation of cigars.

Previous research has shown that microorganisms such as *Bacillus* and *Corynebacterium* are commonly inoculated during the fermentation of CTLs. *Bacillus* species are capable of degrading macromolecular compounds, such as starch and cellulose in CTLs. On the other hand, *Corynebacterium* produces aldehydes and ketones, which play a key role in enhancing the flavor profile of cigars (Yao et al., 2022; Zheng et al., 2022). These studies have demonstrated that the inoculation of bacteria during the fermentation of CTLs accelerates flavor maturation and transformation, significantly improving sensory quality. While *Staphylococcus* are known to be the predominant bacteria involved in CTL fermentation, no studies have yet validated the specific effect of *Staphylococcus nepalensis* on this process. In the present study, we conducted a heatmap analysis to examine the bacteria-flavor association during the cigar fermentation process. This analysis revealed that *Staphylococcus nepalensis* was positively correlated with carotenoid degradation products such as megastigmatrienone, β-damascone, and isophorone, as well as other compounds like benzyl alcohol and solanesone. Based on these findings, we hypothesize that *Staphylococcus nepalensis* contributes to the synthesis and accumulation of these flavor-active substances. To test this hypothesis, *Staphylococcus nepalensis* was isolated and used for the inoculated fermentation of CTLs. Both the inoculated and naturally fermented CTLs were subsequently analyzed for their flavor profiles, physicochemical properties, and sensory quality.

The results indicate that inoculating *Staphylococcus nepalensis* into the fermentation process not only significantly enhanced the flavor of CTLs but also improved the sensory scores of the cigars. Notably, these compounds reached their peak levels after 14 days of fermentation with *Staphylococcus nepalensis* (N-14) and subsequently degraded as fermentation progressed. This is primarily due to the reduction of aroma precursors in the later stages of fermentation and the volatilization of aromas at higher fermentation temperatures (Zhang et al., 2020). In contrast, the levels of these compounds in the CK group gradually increased, reaching their highest concentrations after 21 days of fermentation (CK-21) (Figure 6A). This suggests that the addition of *Staphylococcus nepalensis* accelerated the conversion of macromolecules and the accumulation of aromatic compounds (Hu et al., 2022). Levels of farnesyl acetone, dihydroactinidiolide, and megastigmatrienone significantly increased by day 14 of fermentation (N-14). This can be attributed to the presence of *Staphylococcus nepalensis*, which enhances the growth and metabolic efficiency of microorganisms, thereby promoting the degradation of aromatic substances such as carotenoids (Song et al., 2024). Throughout the cigar fermentation process, the chemical composition of the cigars underwent significant changes. Polysaccharides like starch were converted into sugars (Gong et al., 2023). The total sugar content in the CK group continued to rise, while in the N groups, it peaked and then declined on day 7, indicating that inoculated fermentation accelerated the metabolism of total sugars. During the first 7 days of fermentation, increased microbial activity led to a higher consumption rate of reducing sugars than their production rate, resulting in an increase in total sugars and a decrease in reducing sugars. Reducing sugars and amino acids serve as precursors for the Maillard reaction, which occurs between days 7 and 21 of fermentation. During this period, reducing sugars are utilized to form Maillard reaction products, which are then further degraded to generate aroma compounds (Roemer et al., 2012). Their overall increase in total nitrogen content may be due to an increase in amino acid content during the fermentation process, which requires further experiments to prove. However, within certain concentration ranges, the content of conventional chemicals and aroma substances does not necessarily correlate directly with the quality of CTLs. To assess this, professional sippers were invited to perform sensory evaluations. The results showed that the addition of *Staphylococcus nepalensis* significantly improved the sensory evaluation compared to both the natural fermentation group (CK) and the unfermented group (control). Notably, irritation was reduced, the aftertaste became more pleasant, and sweetness increased, which was linked to the higher total sugar content and the degradation of nicotine (Xue et al., 2023). Furthermore, due to the inoculated fermentation time, wood waste gases were also generated. While 21 days of fermentation yielded some sensory improvements, it was not as effective as the 14-day fermentation period. Therefore, 14 days is considered the optimal fermentation time.

In microbial inoculation fermentation of CTLs, the aroma-causing substances will increase and then decrease with the fermentation time, and the macromolecular compounds such as total sugar and total nitrogen will also be degraded (Jia et al., 2024). In the microbial inoculation fermentation of CTLs, the levels of aroma-causing substances initially increase and then decrease over time, while macromolecular compounds such as total sugars and total nitrogen also undergo degradation. If the fermentation period is too long, it may hinder the improvement of CTL quality, making the control of fermentation time particularly crucial. Different fermentation methods and control techniques are proprietary to each cigar brand and kept confidential. Research on the application of direct-pitch inoculation fermentation in cigars is limited and lacks a systematic approach. In this study, the optimal fermentation period for cigars inoculated with *Staphylococcus nepalensis* was determined to be 14 days. This conclusion was based on sensory evaluations by professional smokers and the measurement of aroma-causing substances in the fermented cigars. Compared to natural fermentation, inoculated fermentation accelerates the maturation of CTLs and the development of flavor shortens the fermentation cycle, and reduces time-related costs. These findings contribute valuable theoretical insights to the study of cigar fermentation processes.

## 5 Conclusion

In conclusion, this study elucidates the bacterial community structure and succession patterns during the fermentation of CTLs through amplicon sequencing. The relative abundance of *Staphylococcus* increased throughout the fermentation process, reaching 80% at the end of fermentation (EFS), thereby dominating the bacterial community. Microbial-flavor correlation analysis revealed that *Staphylococcus nepalensis* contributes to the production of flavor compounds, such as megastigmatrienone, solanesone. Based on these findings, *in vitro* isolation and biologically enhanced fermentation of *Staphylococcus nepalensis* were conducted, and the fermentation performance of the strain was evaluated. The results demonstrated that *Staphylococcus nepalensis* not only significantly increased the content of flavor compounds in CTLs and shortened the fermentation period, but also reduced cigar irritation, improved the aftertaste, enhanced sweetness, and overall sensory quality. This study provides insights into the role of *Staphylococcus nepalensis* as a fermenting agent, affecting the chemical composition, volatile aroma compounds, and sensory quality of CTLs. The findings offer valuable theoretical and technical support for the future development and application of microbial agents in fermentation processes.

## Data Availability

The original contributions presented in the study are included in the article/supplementary material, further inquiries can be directed to the corresponding author.

## References

[ref1] Alpert H. Agaku I. Connolly G. (2015). A study of pyrazines in cigarettes and how additives might be used to enhance tobacco addiction. Tob. Control. 25, 444–450. doi: 10.1136/tobaccocontrol-2014-051943, PMID: 26063608 PMC4941150

[ref2] Aro-Aro J. Purevdorj N.-O. Tsuji K. Shimada K. Fukushima M. Sekikawa M. (2010). The effect of starter culture on proteolytic changes and amino acid content in fermented sausage. Food Chem. 119, 279–285. doi: 10.1016/j.foodchem.2009.06.025

[ref3] Ashraf-Khorasani M. Coleman W. Dube M. Taylor L. (2018). Synthesis of pyrazines using sugar derived from tobacco cellulose and hydrolyzed tobacco F1 protein as an amino acid source. Contrib. Tob. Nicotine Res. 28, 103–111. doi: 10.2478/cttr-2018-0011, PMID: 39513035

[ref4] Banožić M. Jokic S. Ackar D. Blažić M. Šubarić D. (2020). Carbohydrates-key players in tobacco aroma formation and quality determination. Molecules 25:1734. doi: 10.3390/molecules25071734, PMID: 32283792 PMC7181196

[ref5] Gong Y. Li J. Deng X. Chen Y. Chen S. Huang H.. (2023). Application of starch degrading bacteria from tobacco leaves in improving the flavor of flue-cured tobacco. Front. Microbiol. 14:1211936. doi: 10.3389/fmicb.2023.1211936, PMID: 37440887 PMC10335769

[ref6] He P. Wang Y. Geng Z. Fang S. Qiu J. Qiu J. (2023). Study on key volatile aroma components of cigar filler from different producing areas. Chin. Tob. Sci. 44, 92–99. doi: 10.13496/j.issn.1007-5119.2023.01.014

[ref7] Hu W. Cai W. Zheng Z. Liu Y. Luo C. Xue F.. (2022). Study on the chemical compositions and microbial communities of cigar tobacco leaves fermented with exogenous additive. Sci. Rep. 12:19182. doi: 10.1038/s41598-022-23419-y, PMID: 36357535 PMC9649726

[ref8] Hu Y. Chen Q. Wen R. Wang Y. Qin L. Kong B. (2019). Quality characteristics and flavor profile of Harbin dry sausages inoculated with lactic acid bacteria and *Staphylococcus xylosus*. LWT 114:108392. doi: 10.1016/j.lwt.2019.108392

[ref9] Huang R. Crowther T. Sui Y. Sun B. Liang Y. (2021). High stability and metabolic capacity of bacterial community promote the rapid reduction of easily decomposing carbon in soil. Commun. Biol. 4:1376. doi: 10.1038/s42003-021-02907-3, PMID: 34880408 PMC8654823

[ref10] Jia Y. Guo S. Zhang Q. Hu W. Zhu B. Li D. (2024). Study on single factor optimization of industrial fermentation conditions for domestic cigar tobacco leaves. Acta Tabac. Sin. 30, 105–115. doi: 10.16472/j.chinatobacco.2022.135

[ref11] Jia Y. Liu Y. Hu W. Cai W. Zheng Z. Luo C.. (2023). Development of *Candida* autochthonous starter for cigar fermentation via dissecting the microbiome. Front. Microbiol. 14:1138877. doi: 10.3389/fmicb.2023.1138877, PMID: 36910204 PMC9998997

[ref12] Jiang Y. Gong W. Xian Z. Xu W. Hu J. Ma Z.. (2023). 16S full-length gene sequencing analysis of intestinal flora in breast cancer patients in Hainan Province. Mol. Cell. Probes 71:101927. doi: 10.1016/j.mcp.2023.101927, PMID: 37595804

[ref13] Leroy S. Vermassen A. Ras G. Talon R. (2017). Insight into the genome of *Staphylococcus xylosus*, a ubiquitous species well adapted to meat products. Microorganisms 5:52. doi: 10.3390/microorganisms5030052, PMID: 28850086 PMC5620643

[ref14] Li X. Kang C. Bin J. (2022). Research Progress on the Degradation Law of Latent Flavor in Tobacco Leaf Alcoholization. Anhui Agric. Sci. 50, 21–26. doi: 10.3969/j.issn.0517-6611.2022.13.007

[ref15] Li Y. Luo X. Guo H. Bai J. Xiao Y. Fu Y.. (2023). Metabolomics and metatranscriptomics reveal the influence mechanism of endogenous microbe (*Staphylococcus succinus*) inoculation on the flavor of fermented chili pepper. Int. J. Food Microbiol. 406:110371. doi: 10.1016/j.ijfoodmicro.2023.110371, PMID: 37659279

[ref16] Li J. Zhao Y. Qin Y. Shi H. (2020). Influence of microbiota and metabolites on the quality of tobacco during fermentation. BMC Microbiol. 20:356. doi: 10.1186/s12866-020-02035-8, PMID: 33213368 PMC7678276

[ref17] Liu T. Guo S. Wu C. Zhang R. Zhong Q. Shi H.. (2022). Phyllosphere microbial community of cigar tobacco and its corresponding metabolites. Front. Microbiol. 13:1025881. doi: 10.3389/fmicb.2022.1025881, PMID: 36439836 PMC9691965

[ref18] Liu Z. Wang Z. Lv X. Zhu X. Chen L. Ni L. (2018). Comparison study of the volatile profiles and microbial communities of Wuyi Qu and Gutian Qu, two major types of traditional fermentation starters of Hong Qu glutinous rice wine. Food Microbiol. 69, 105–115. doi: 10.1016/j.fm.2017.07.019, PMID: 28941890

[ref19] Liu F. Wu Z. Zhang X. Xi G. Zhao Z. Lai M.. (2021). Microbial community and metabolic function analysis of cigar tobacco leaves during fermentation. Microbiologyopen 10:e1171. doi: 10.1002/mbo3.1171, PMID: 33970539 PMC8483401

[ref20] Liu A. Yuan K. Li Q. Liu S. Li Y. Tao M.. (2022). Metabolomics and proteomics revealed the synthesis difference of aroma precursors in tobacco leaves at various growth stages. Plant Physiol. Biochem. 192, 308–319. doi: 10.1016/j.plaphy.2022.10.016, PMID: 36288661

[ref9001] Ma X. Zhang Y. Li X. Bi X. Zhang G. (2022). Impacts of salt-tolerant Staphylococcus nepalensis 5-5 on bacterial composition and biogenic amines accumulation in fish sauce fermentation. Int. J. Food Microbiol. 361, 109464. doi: 10.1016/j.ijfoodmicro.2021.109464, PMID: 34749187

[ref21] Mindt M. Beyraghdar Kashkooli A. Suarez-Diez M. Ferrer L. Jilg T. Bosch D.. (2022). Production of indole by *Corynebacterium glutamicum* microbial cell factories for flavor and fragrance applications. Microb. Cell Fact. 21:45. doi: 10.1186/s12934-022-01771-y, PMID: 35331232 PMC8944080

[ref22] Orland C. Emilson E. Basiliko N. Mykytczuk N. Gunn J. Tanentzap A. (2018). Microbiome functioning depends on individual and interactive effects of the environment and community structure. ISME J. 13, 1–11. doi: 10.1038/s41396-018-0230-x, PMID: 30042502 PMC6298968

[ref23] Popova V. Ivanova T. Prokopov T. Nikolova M. Stoyanova A. Zheljazkov V. D. (2019). Carotenoid-related volatile compounds of tobacco (*Nicotiana tabacum* L.) essential oils. Molecules 24:3446. doi: 10.3390/molecules24193446, PMID: 31547525 PMC6804150

[ref24] Roemer E. Schorp M. Piadé J.-J. Seeman J. Leyden D. Haussmann H.-J. (2012). Scientific assessment of the use of sugars as cigarette tobacco ingredients: a review of published and other publicly available studies. Crit. Rev. Toxicol. 42, 244–278. doi: 10.3109/10408444.2011.650789, PMID: 22263649 PMC3296517

[ref25] Sánchez Mainar M. Xhaferi R. Samapundo S. Devlieghere F. Leroy F. (2016). Opportunities and limitations for the production of safe fermented meats without nitrate and nitrite using an antibacterial *Staphylococcus sciuri* starter culture. Food Control 69, 267–274. doi: 10.1016/j.foodcont.2016.04.056

[ref26] Silinski M. Uenoyama T. Coleman D. Blake J. Thomas B. Marusich J.. (2020). Analysis of nicotine and non-nicotine tobacco constituents in aqueous smoke/aerosol extracts by UHPLC and ultraperformance convergence chromatography-tandem mass spectrometry. Chem. Res. Toxicol. 33, 2988–3000. doi: 10.1021/acs.chemrestox.0c00312 33226218

[ref27] Slaghenaufi D. Perello M.-C. Marchand S. Revel G. (2016). Quantification of megastigmatrienone, a potential contributor to tobacco aroma in spirits. Food Chem. 203, 41–48. doi: 10.1016/j.foodchem.2016.02.034, PMID: 26948587

[ref28] Song W. Chen X. Yu J. Qiao J. Yang J. Chen X.. (2024). Effects of *Bacillus altitudinis* inoculants on cigar tobacco leaf fermentation. Front. Bioeng. Biotechnol. 12:1417601. doi: 10.3389/fbioe.2024.1417601, PMID: 39045536 PMC11264575

[ref29] Tan S. Zeng Y. Zeng J. (2023). Research Progress of Fermentation Effect on Quality of Cigar Tobacco Leaves. Anhui Agric. Sci. 51, 16–19+28. doi: 10.3969/j.issn.0517-6611.2023.18.004

[ref900101] Uckun O. Selli S. (2017). Characterization of key aroma compounds in a representative aromatic extracts from citrus and astragalus honeys based on aroma extract dilution analyses. J. Food Meas. Charact. 11, 512–522. doi: 10.1007/s11694-016-9418-9, PMID: 33512154

[ref30] Vu A. T. Hassink M. D. Taylor K. M. McGuigan M. Blasiole A. Valentin-Blasini L.. (2021). Volatile organic compounds in mainstream smoke of sixty domestic little cigar products. Chem. Res. Toxicol. 34, 704–712. doi: 10.1021/acs.chemrestox.0c00215, PMID: 33512154 PMC10042296

[ref31] Wang H. Xu J. Liu Q. Xia X. Sun F. Kong B. (2022). Effect of the protease from *Staphylococcus carnosus* on the proteolysis, quality characteristics, and flavor development of Harbin dry sausage. Meat Sci. 189:108827. doi: 10.1016/j.meatsci.2022.108827, PMID: 35429823

[ref32] Wu Q. Peng Z. Pan Y. Liu L. Li L. Zhang J.. (2023a). Interaction analysis of tobacco leaf microbial community structure and volatiles flavor compounds during cigar stacking fermentation. Front. Microbiol. 14:1168122. doi: 10.3389/fmicb.2023.1168122, PMID: 37637131 PMC10457113

[ref33] Wu Q. Shi Y. Li L. Peng Z. Tan Z. Liu L.. (2023b). *In situ* screening of carotenoid degrading strains and the application in improving quality and aroma of cigar. Biotechnol. Bull. 39, 192–201. doi: 10.13560/j.cnki.biotech.bull.1985.2023-0258

[ref34] Xue F. Yang J. Luo C. Li D. Shi G. Song G.. (2023). Metagenomic insight into the biodegradation of biomass and alkaloids in the aging process of cigar. Bioresour. Bioprocess. 10:45. doi: 10.1186/s40643-023-00667-y, PMID: 38647787 PMC10992288

[ref35] Yang J. Mao D. Chen Z. Sun Z. Hao H. Jia C.. (2016). Determination of odor active values of megastigmatrienone in cigarette mainstream smoke by GC-MS/O. Acta Tabac. Sin. 22, 11–17. doi: 10.16472/j.chinatobacco.2015.491

[ref36] Yao L. Huang C. Ding J. Zhang T. Yu J. Yang C.. (2022). Application of yeast in plant-derived aroma formation from cigar filler leaves. Front. Bioeng. Biotechnol. 10:1093755. doi: 10.3389/fbioe.2022.1093755, PMID: 36619396 PMC9815610

[ref37] Ye C. Li L. He C. Li D. (2021). Structure and diversity analysis of microbial communities in cigar products by high-throughput sequencing technology. Tob. Sci. Technol. 54, 1–9. doi: 10.16135/j.issn1002-0861.2020.0610

[ref38] Yin F. Eric K. Song S. Emmanuel D. Lin S. Heping C.. (2018). Contribution of tobacco composition compounds to characteristic aroma of Chinese faint-scent cigarettes through chromatography analysis and partial least squares regression. J. Chromatogr. B 1105, 217–227. doi: 10.1016/j.jchromb.2018.12.001, PMID: 30611933

[ref39] Yun L. (2016). Rich collect and application of solanone in tobacco. Chem. Intermed., 9, 32–33.

[ref40] Yun J. Cui C. Zhang S. Zhu J. Peng C. Cai H.. (2021). Use of headspace GC/MS combined with chemometric analysis to identify the geographic origins of black tea. Food Chem. 360:130033. doi: 10.1016/j.foodchem.2021.130033, PMID: 34023716

[ref9002] Yu J. Lu K. Dong X. Xie W.. (2022). Virgibacillus sp. SK37 and Staphylococcus nepalensis JS11 as potential starters to improve taste of shrimp paste. LWT, 115:112657. doi: 10.1016/j.lwt.2021.112657, PMID: 34023716

[ref41] Zhang L. Y. Mai J. Shi J. F. Ai K. B. He L. Zhu M. J.. (2024). Study on tobacco quality improvement and bacterial community succession during microbial co-fermentation. Ind. Crop. Prod. 208:117889. doi: 10.1016/j.indcrop.2023.117889

[ref42] Zhang R. Su Q. Yang C. Chen F. (2020). Effect of stacking fermentation time on quality of Wuzhishan cigar wrapper tobacco leaves. Shandong Agric. Sci. 52, 57–61. doi: 10.14083/j.issn.1001-4942.2020.04.010

[ref43] Zhang W. Xiao Z. Gu Z. Deng X. Liu J. Luo X.. (2024). Fermentation-promoting effect of three salt-tolerant Staphylococcus and their co-fermentation flavor characteristics with *Zygosaccharomyces rouxii* in soy sauce brewing. Food Chem. 432:137245. doi: 10.1016/j.foodchem.2023.137245, PMID: 37657348

[ref44] Zhang L. Xiong S. Du T. Xiao M. Peng Z. Xie M.. (2023). Effects of microbial succession on the dynamics of flavor metabolites and physicochemical properties during soy sauce koji making. Food Biosci. 53:102636. doi: 10.1016/j.fbio.2023.102636

[ref45] Zhang Q. Yang S. Yang Z. Zheng T. Li P. Zhou Q.. (2023a). Effects of a novel microbial fermentation medium produced by *Tremella aurantialba* SCT-F3 on cigar filler leaf. Front. Microbiol. 14:1267916. doi: 10.3389/fmicb.2023.1267916, PMID: 37808308 PMC10556473

[ref46] Zhang G. Yao H. Zhao G. Wu Y. Xia H. Li Y.. (2023). Metabolomics reveals the effects producing region and fermentation stage on substance conversion in cigar tobacco leaf. Chem. Biol. Technol. Agric. 10:66. doi: 10.1186/s40538-023-00444-1

[ref47] Zhang Q. Zheng T. Yang Z. Yang S. Cai W. Li P.. (2023b). Analysis of the structure and metabolic function of microbial community in cigar tobacco leaves in agricultural processing stage. Front. Microbiol. 14:1230547. doi: 10.3389/fmicb.2023.1230547, PMID: 37637128 PMC10448963

[ref48] Zhao S. Wu Z. Lai M. Zhao M. Lin B. (2022). Determination of optimum humidity for air-curing of cigar tobacco leaves during the browning period. Ind. Crop. Prod. 183:114939. doi: 10.1016/j.indcrop.2022.114939

[ref49] Zheng T. Zhang Q. Wu Q. Li D. Wu X. Li P.. (2022). Effects of inoculation with *Acinetobacter* on fermentation of cigar tobacco leaves. Front. Microbiol. 13:911791. doi: 10.3389/fmicb.2022.911791, PMID: 35783443 PMC9248808

[ref50] Zhou J. (2021). Dynamic characteristics and co-occurrence patterns of microbial community in tobacco leaves during the 24-month aging process. Ann. Microbiol. 71:9. doi: 10.1186/s13213-021-01620-0

[ref51] Zong P. Hu W. Huang Y. An H. Zhang Q. Chai Z.. (2023). Effects of adding cocoa fermentation medium on cigar leaves in agricultural fermentation stage. Front. Bioeng. Biotechnol. 11:1251413. doi: 10.3389/fbioe.2023.1251413, PMID: 37662435 PMC10469782

